# Supplementary material for: Mediterranean Diet and Risk of Dementia and Alzheimer’s Disease in the EPIC-Spain Dementia Cohort Study
Source: Nutrients. 2021 Feb 22;13(2):700. doi: 10.3390/nu13020700 (PMC7927039; doi:10.3390/nu13020700)
Supplement: Supplementary file 1 [file nutrients-13-00700-s001.pdf]

Supplementary data

"Mediterranean diet and risk of dementia and Alzheimer's disease in the European Prospective Investigation into Cancer and nutrition-Spain Dementia Cohort study".

Andreu-Reinón et al.

Figure S1.

Figure S2.

Figure S3.

Figure S4.

Figure S5.

Figure S6.

Table S1.

References.

Figure S1. Flow chart of study participants.

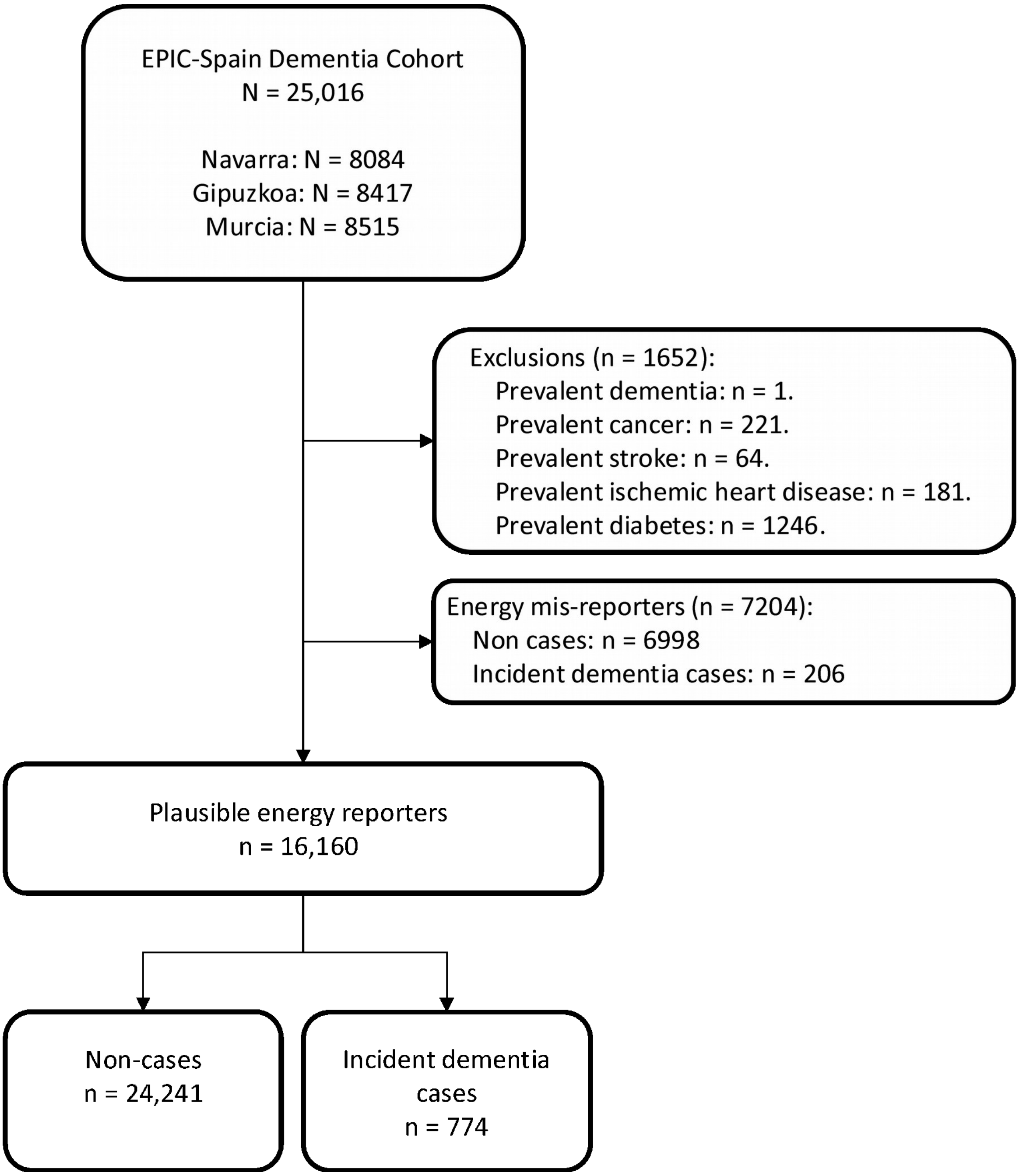

Figure S2. Effect of an item-by-item exclusion of components of the Mediterranean Diet score (rMED) on the overall estimates of dementia risk.

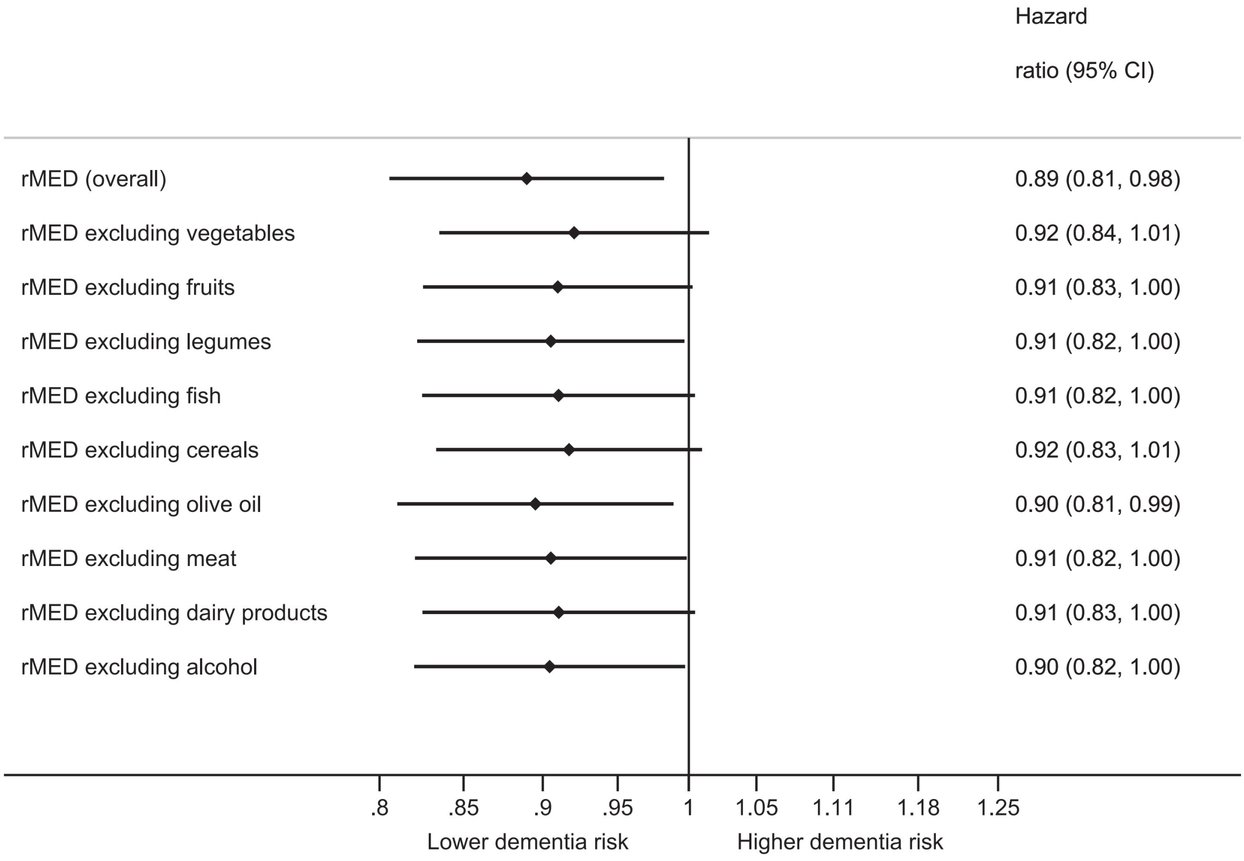

Exclusion of single rMED components had little effect on overall estimates. Hazard ratios of dementia per 1 standard deviation change in the rMED were computed using Cox proportional hazards regression models stratified by center and age (in 5-year categories), and adjusted by sex, education, energy intake, smoking, BMI category, elevated waist circumference, household and recreational physical activities, hypertension (self-reported), hyperlipidemia (self-reported), coffee and tea consumption (combined), and intake (in g/day per 2000 kcal) of potatoes, eggs, and cakes and biscuits. Age was defined as the underlying time scale.

Figure S3. Sensitivity analyses of dementia risk under different multivariable models (M1-M6) in the EPIC-Spain Dementia Cohort study (N = 16,160).

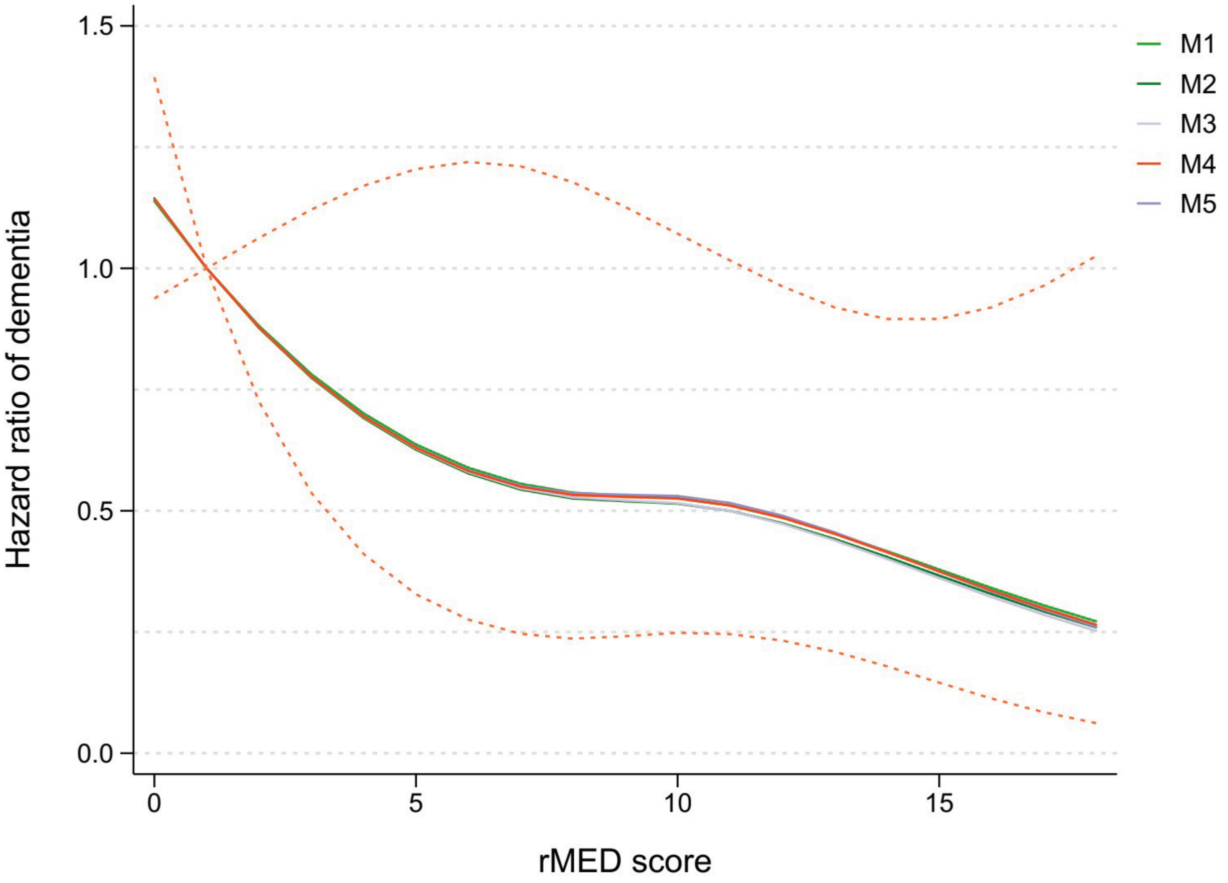

Results were robust to a series of sensitivity analysis. Models M1-M5 imply different adjustment levels or exclusion schemes, where M4 denotes the final multivariable model of the main analysis and it is represented in orange with 95% confidence intervals (dotted lines). M1: basic model (rMED, no covariates). M2: M1 + sex, education, and energy intake. M3: M2 + sex, education, energy intake, smoking, BMI, elevated waist circumference, physical activity (household and recreational), hypertension, and hyperlipidemia. M4 (main analysis): sex, education, energy intake, smoking, BMI, elevated waist circumference, physical activity (household and recreational), hypertension, hyperlipidemia, coffee and tea consumption, and intake of potatoes, eggs, and cakes and biscuits. M5: M4, after exclusion of the first 5 years of follow-up. Cox regression models with age as the time scale, stratified by center and age categories (5-year groups), following a restricted cubic spline transformation of the Mediterranean Diet score.

Figure S4. Hazard ratio of dementia in relation to alternative Mediterranean Diet scores in the EPIC-Spain Dementia Cohort study (N = 16,160).

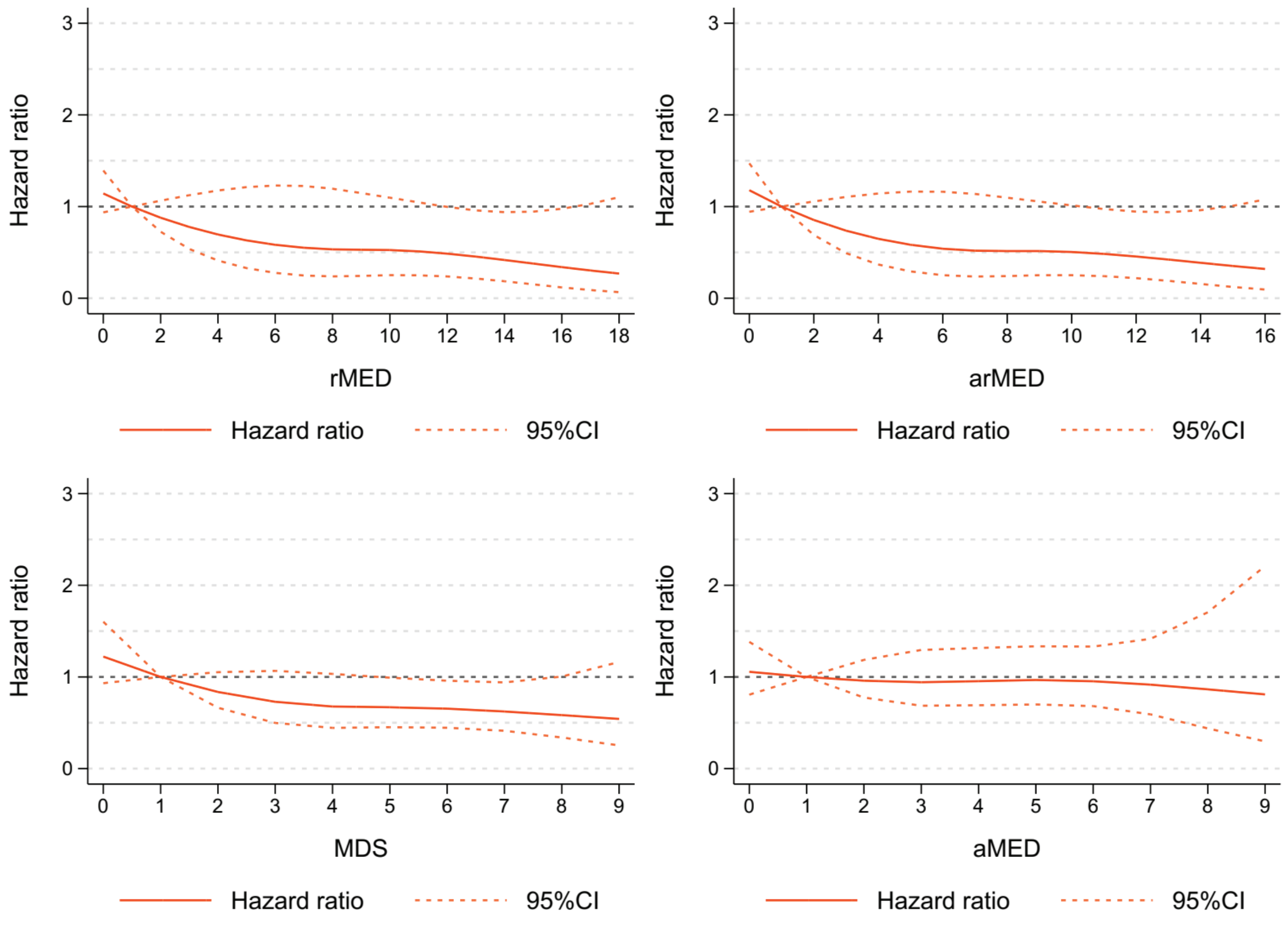

The association effect size for Mediterranean Diet and dementia risk varied depending on the score used. rMED and arMED showed the largest effects in our study, whereas estimates were atenuated for the MDS, and null for the aMED score. Cox regression models with age as the time scale, stratified by center and age categories (5-year groups), following a restricted cubic spline transformation of the exposure variables. Hazard ratios (solid lines) and 95% confidence intervals (dotted lines) of dementia from multivariable models were plotted against several Mediterranean Diet scores, which are different adaptations of the original Mediterranean Diet Score developed by Trichopoulou *et al*. (1): the relative Mediterranean Diet score (rMED) by Buckland *et al*. (2), the adapted relative Mediterranean Diet score (arMED) by Buckland *et al*. (3), the Mediterranean Diet Score (MDS) by Trichopoulou *et al*. (4), and the alternate Mediterranean Diet Index (aMED) by Fung *et al*. (5). Each score was defined as follows: i) rMED (0-18 points), intake (in g/day per 2000 kcal) of 8 dietary components was categorized into tertiles, and given a score of 0 to 2 to increasing tertiles of intake of supposed beneficial components (vegetables, fruits and nuts, legumes, fish and seafood, cereals, and olive oil) and a reverse scoring for supposed detrimental ones (meat, and dairy products); for alcohol, 2 points were given for intakes within a given range (10-50 g/day for men, 5-25 g/day for women), and 0 otherwise. ii) arMED (0-16 points), an adaptation of the rMED score, excluding alcohol from the definition of the index. Iii) MDS (0-9 points), 1 point was given to intakes at or above the sex-specific median intake of beneficial components (vegetables, legumes, fruits and nuts, cereals, fish and shellfish, and ratio of monounsaturated to saturated lipids), and 0 points to intakes

below the median; for detrimental components (meat and poultry, and dairy products) a score of 1 was given to intakes below the median, and 0 to intakes at or above this cutoff; for ethanol, 1 point was given to intakes within 10-50 g/d (men) or 5-25 g/day (women), and 0 points to consumption outside this range. iv) aMED (0-9 points), intakes at or above the se-specific median of vegetables (excluding potatoes), legumes, fruits and fruit juices, fish and shellfish, and monounsaturated-to-polyunsaturated fats ratio scored 1 point, whereas intakes below the corresponding median scored 0 points. For nuts and whole-grain products (whole-bread), 1 point was given to any intake above 0 (given the low cosumption in the cohort at the date of recruitment), and 0 to non-consumers. Ethanol intake scored 1 point within the defined range of 10-50 g/d (men) or 5-25 g/day (women) and 0 otherwise.

Figure S5. Hazard ratio of dementia, overall and by subtypes, according to Mediterranean Diet scores in the EPIC-Spain Dementia Cohort study (N = 16,160).

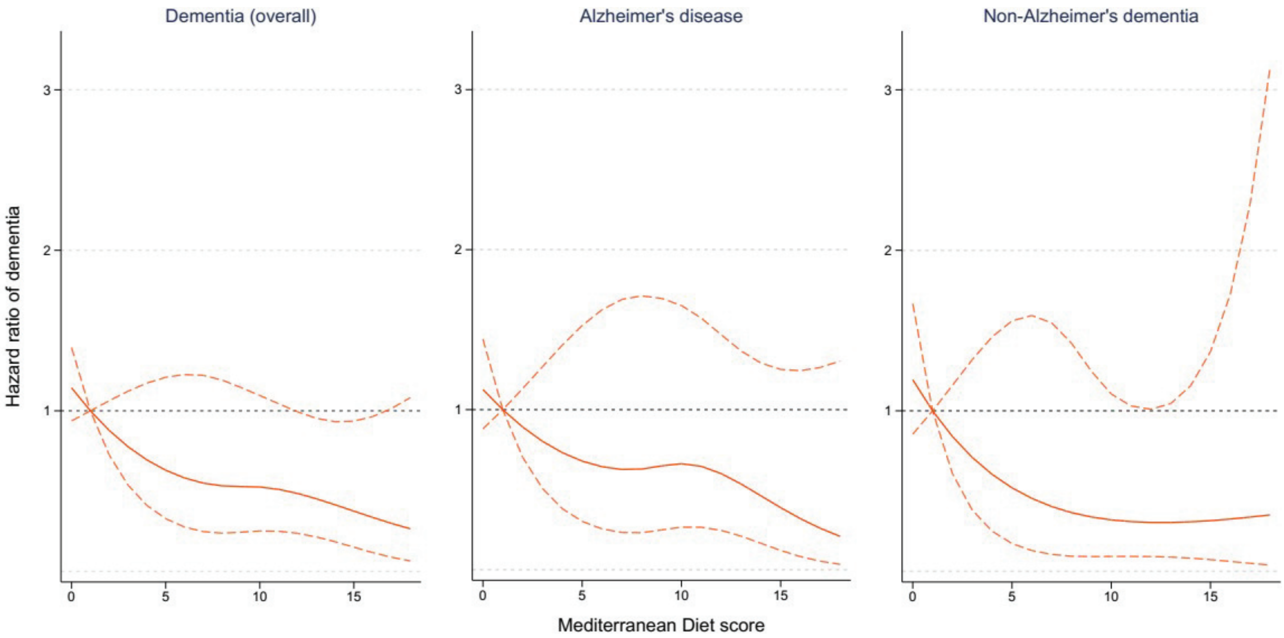

Hazard ratios of dementia were estimated using Cox proportional hazards regression models, with age as the time scale, stratified by center and age (in 5-year categories), and adjusted by sex, education, energy intake, smoking, BMI category, elevated waist circumference, household and recreational physical activities, hypertension (self-reported), hyperlipidemia (self-reported), coffee and tea consumption (combined), and intake (in g/day per 2000 kcal) of potatoes, eggs, and cakes and biscuits. In women, models were further adjusted by menopausal status, oral contraceptive use, and hormone replacement therapy. Risk of each dementia-related outcome was modelled following a restricted cubic spline transformation of the rMED variable with 3 degrees of freedom (knots were placed at the 33rd and 67th percentiles).

Figure S6. Effect of dietary energy mis-reporting on hazard ratio estimates of dementia in the EPIC-Spain Dementia Cohort study (N = 16,160).

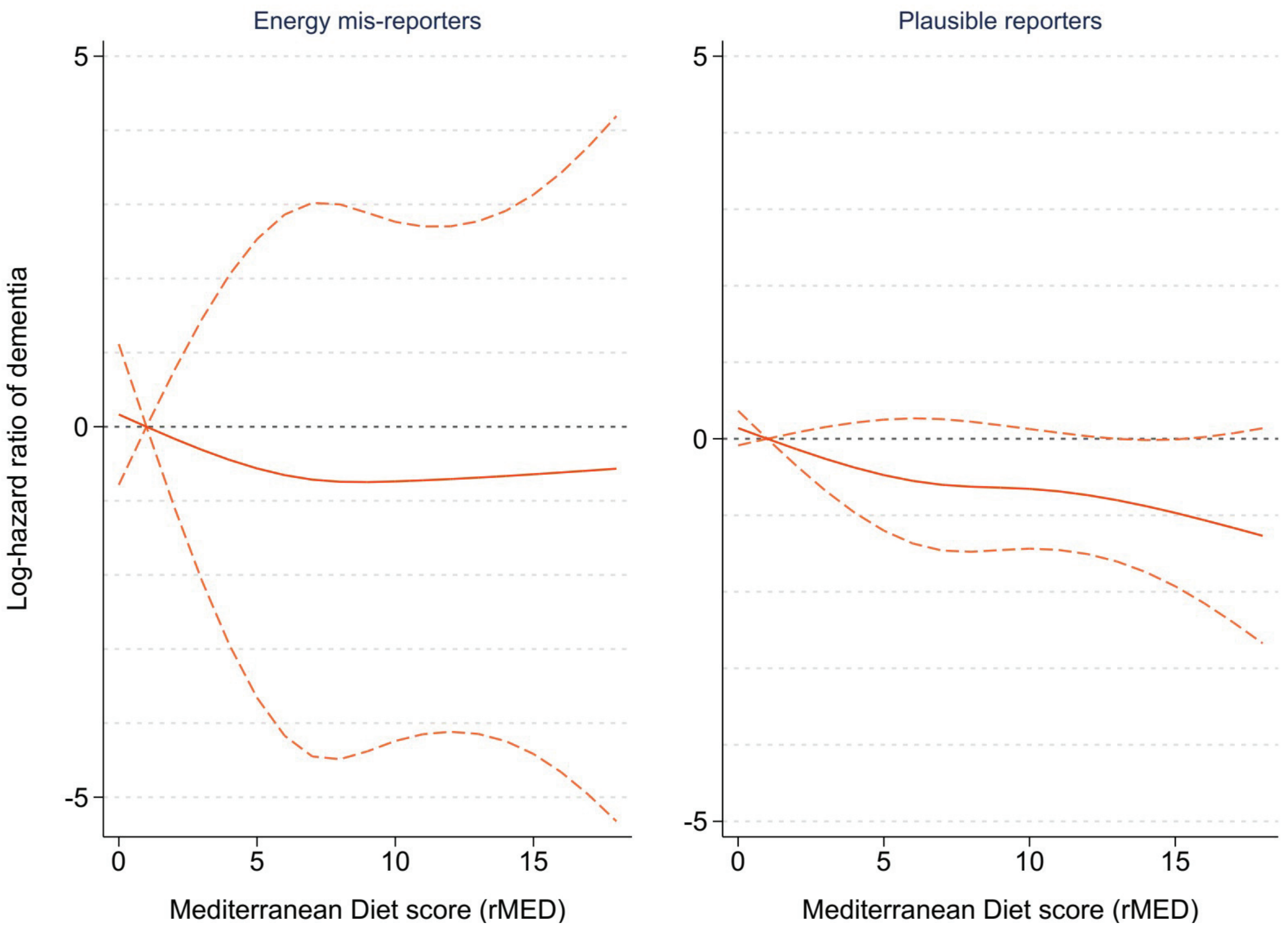

Plausibility of energy intake was established as described by Mendez *et al*. (6) using the predicted total energy expenditure (pTEE) method. pTEE was calculated using the Dietary Reference Intakes prediction equations (7), and mis-reporters were defined as those beyond ±30% deviation of the ratio of reported intakes to estimated requirements (rEI:pTEE). Hazard ratios of dementia were estimated using Cox proportional hazards regression models, with age as the time scale, stratified by center and age (in 5-year categories), and adjusted by sex, education, energy intake, smoking, BMI category, elevated waist circumference, household and recreational physical activities, hypertension (self-reported), hyperlipidemia (self-reported), coffee and tea consumption (combined), and intake (in g/day per 2000 kcal) of potatoes, eggs, and cakes and biscuits. Dementia risk was modelled following a restricted cubic spline transformation of the rMED variable with 3 degrees of freedom (knots were placed at the 33th and 67th percentiles).

Table S1. Baseline characteristics of study participants, by categories of the Mediterranean Diet score (N = 16,160).

	rMED score			
	Low	Medium	High	*P*
Age (y)	48.0 (7.8)	49.0 (7.8)	50.1 (7.7)	**< 0.001**
Female sex	2,196 (68.7%)	4,862 (57.9%)	2,181 (47.8%)	**< 0.001**
Low educational level[1]	2,343 (73.2%)	6,035 (71.9%)	3,244 (71.1%)	0.115
Current smoker	1,014 (31.7%)	2,378 (28.3%)	1,248 (27.4%)	**< 0.001**
Overweight or obese	2,404 (75.2%)	6,507 (77.5%)	3,630 (79.6%)	**< 0.001**
Elevated waist circumference[2]	1,336 (41.8%)	3,566 (42.5%)	1,885 (41.3%)	0.427
Leisure-time physical activity (MET·h/week)[3]	99.3 (58.2)	91.7 (58.0)	86.2 (55.1)	**< 0.001**
Energy intake (kcal/day)	2073.4 (475.2)	2112.2 (492.6)	2148.6 (471.9)	**< 0.001**
Potatoes (g/day per 2000 kcal)	69.9 (42.8)	76.8 (42.7)	83.4 (45.2)	**< 0.001**
Vegetables (g/day per 2000 kcal)	186.1 (116.9)	259.8 (144.2)	322.8 (145.3)	**< 0.001**
Fruits (g/day per 2000 kcal)	238.7 (221.3)	314.4 (226.9)	379.0 (220.6)	**< 0.001**
Legumes (g/day per 2000 kcal)	34.5 (28.1)	46.5 (30.2)	59.7 (33.2)	**< 0.001**
Fish and seafood (g/day per 2000 kcal)	40.3 (27.6)	58.0 (36.8)	77.3 (41.5)	**< 0.001**
Cereals (g/day per 2000 kcal)	155.9 (67.9)	181.6 (70.0)	205.5 (65.0)	**< 0.001**
Olive oil (g/day per 2000 kcal)	12.0 (11.3)	19.4 (13.9)	26.4 (12.6)	**< 0.001**
Nuts and seeds (g/day per 2000 kcal)	3.0 (7.5)	3.1 (7.3)	3.5 (7.6)	**< 0.001**
Meat (g/day per 2000 kcal)	141.7 (48.9)	125.4 (47.2)	104.1 (41.8)	**< 0.001**
Dairy products (g/day per 2000 kcal)	371.0 (202.5)	278.1 (174.5)	197.7 (132.7)	**< 0.001**
Eggs (g/day per 2000 kcal)	26.9 (19.4)	26.0 (18.1)	23.7 (17.0)	**< 0.001**
Coffee and tea (ml/day)	138.6 (134.1)	121.2 (123.1)	109.5 (115.3)	**< 0.001**
Alcohol (g/day)	11.7 (23.3)	13.9 (20.5)	15.4 (15.9)	**< 0.001**
Mediterranean diet score (rMED)	5.0 (1.1)	8.6 (1.1)	12.2 (1.3)	**< 0.001**
Women only (N = 9,239)				
Post-menopausal	748 (34.1%)	1,767 (36.3%)	879 (40.3%)	**< 0.001**
Ever use of hormonal replacement therapy	234 (11.0%)	466 (9.9%)	231 (10.8%)	0.271
Ever use of oral contraceptives	944 (43.0%)	2,016 (41.5%)	883 (40.5%)	0.231

Numbers are frequencies or means and standard deviations.

[1] Primary studies or less

[2] Waist circumference ≥102 cm (men) or ≥88 cm (women).

[3] Sum of recreational and household physical activities.

References

1. Trichopoulou, A., Kouris-Blazos, A., Wahlqvist, M. L., Gnardellis, C., Lagiou, P., Polychronopoulos, E., Vassilakou, T., Lipworth, L. & Trichopoulos, D. Diet and overall survival in elderly people. *BMJ* **311,** 1457–60 (1995).

2. Buckland, G., González, C. A., Agudo, A., Vilardell, M., Berenguer, A., Amiano, P., Ardanaz, E., Arriola, L., Barricarte, A., Basterretxea, M., Chirlaque, M. D., Cirera, L., Dorronsoro, M., Egües, N., Huerta, J. M., Larrañaga, N., Marin, P., Martínez, C., Molina, E., Navarro, C., Quirós, J. R., Rodriguez, L., Sanchez, M.-J., Tormo, M.-J. & Moreno-Iribas, C. Adherence to the Mediterranean diet and risk of coronary heart disease in the Spanish EPIC Cohort Study. *Am. J. Epidemiol.* **170,** 1518–29 (2009).

3. Buckland, G., Travier, N., Cottet, V., González, C. a., Luján-Barroso, L., Agudo, A., Trichopoulou, A., Lagiou, P., Trichopoulos, D., Peeters, P. H., May, A., Bueno-de-Mesquita, H. B., Bvan Duijnhoven, F. J., Key, T. J., Allen, N., Khaw, K. T., Wareham, N., Romieu, I., McCormack, V., Boutron-Ruault, M., Clavel-Chapelon, F., Panico, S., Agnoli, C., Palli, D., Tumino, R., Vineis, P., Amiano, P., Barricarte, A., Rodríguez, L., Sanchez, M. J., Chirlaque, M. D., Kaaks, R., Teucher, B., Boeing, H., Bergmann, M. M., Overvad, K., Dahm, C. C., Tjønneland, A., Olsen, A., Manjer, J., Wirfält, E., Hallmans, G., Johansson, I., Lund, E., Hjartåker, A., Skeie, G., Vergnaud, a. C., Norat, T., Romaguera, D. & Riboli, E. Adherence to the mediterranean diet and risk of breast cancer in the European prospective investigation into cancer and nutrition cohort study. *Int. J. cancer* **132,** 2918–27 (2013).

4. Trichopoulou, A., Costacou, T., Bamia, C. & Trichopoulos, D. Adherence to a Mediterranean Diet and Survival in a Greek Population. *N. Engl. J. Med.* **348,** 2599–2608 (2003).

5. Fung, T. T., McCullough, M. L., Newby, P. K., Manson, J. A. E., Meigs, J. B., Rifai, N., Willett, W. C. & Hu, F. B. Diet-quality scores and plasma concentrations of markers of inflammation and endothelial dysfunction. *Am. J. Clin. Nutr.* **82,** 163–173 (2005).

6. Mendez, M. A., Popkin, B. M., Buckland, G., Schroder, H., Amiano, P., Barricarte, A., Huerta, J.-M., Quirós, J. R., Sánchez, M.-J. & González, C. A. Alternative methods of accounting for underreporting and overreporting when measuring dietary intake-obesity relations. *Am. J. Epidemiol.* **173,** 448–58 (2011).

7. Institute of Medicine. Dietary Reference Intakes for Energy, Carbohydrate, Fiber, Fat, Fatty Acids, Cholesterol, Protein, and Amino Acids (Macronutrients). Washington, DC: The National Academies Press; 2002.
